# Sn Whisker Growth Mitigation by Modifying the Composition of the Solder Alloys: A Brief Review

**DOI:** 10.3390/ma18051130

**Published:** 2025-03-02

**Authors:** Halim Choi, Balázs Illés, Karel Dušek

**Affiliations:** 1 Department of Electronics Technology, Faculty of Electrical Engineering and Informatics, Budapest University of Technology and Economics, 1111 Budapest, Hungary; inertia9192@gmail.com; 2 LTCC Research Group, Łukasiewicz Research Network, Institute of Microelectronics and Photonics, 30701 Kraków, Poland; 3 Department of Electrotechnology, Faculty of Electrical Engineering, Czech Technical University in Prague, 16000 Prague, Czech Republic; dusekk1@fel.cvut.cz

**Keywords:** soldering, Sn whisker, alloy, composite soldering, IMC layer, corrosion

## Abstract

Soldering with Sn alloys has always been the essential assembly step of microelectronics. The conductive Sn whiskers, which can spontaneously grow from soldering surfaces, mean a considerable reliability risk for microelectronics due to possible short circuit formation between the leads of the components. Since their discovery in 1951, thousands of research studies have been conducted to unravel their growth mechanisms and find effective prevention methods against them. Till 2006, the Sn whisker problem was solved and partially forgotten due to the very effective whisker suppression effect of Pb alloying into the solder materials. The lead-free change gave new impetus to the problem, which was further enhanced by the application of new material systems, growing reliability requirements, and accelerating miniaturization in the 21st century. Our review would like to give an overview of the Sn whisker’s history from the beginning till the latest results, focusing on the suppression solutions by the modification of the solder alloy compositions. Recently, promising results have been reached by alloying Bi and In, which are metals that are the focus of low-temperature soldering, and by composite solders.

## 1. Introduction

### 1.1. Early History of Tin Whiskers

**Figure 1:**
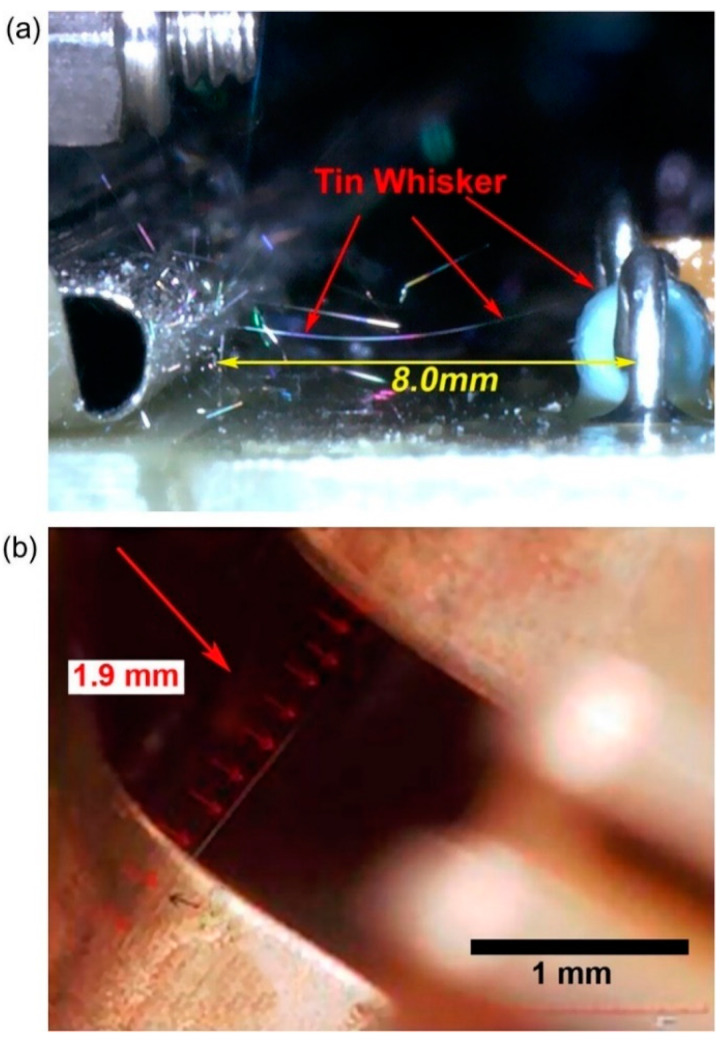
Sn whiskers: (**a**) In a card cage of a space shuttle (photo courtesy of the NASA Shuttle Logistics Depot (NSLD)). (**b**) In an accelerator pedal position sensor of a Toyota Camry [3].

Metal whiskers are conductive filaments that can grow spontaneously on several metal surfaces. The first formation of metal whiskers (from cadmium, Cd) was reported in the 1940s. They grew from electroplated cadmium and shortcut adjacent electrodes in capacitors [1]. In 1948, the Bell Telephone Company experienced a failure in a channel filter applied to maintain frequency bands in a telephone transmission line. The investigation of the failure revealed that Cd whisker growth caused the mall function of the channel filter. The Bell laboratory began a series of long-term, wide-scope research about whisker formation. The first results were reported by Compton et al. in 1951 [2]. These studies revealed that whisker formation does occur naturally but not exclusively in Cd electroplating. Whisker formation was also observed on electroplated zinc, tin, silver, and aluminum cast alloys. However, Sn whiskers received the most attention in the past decades (Figure 1) since Sn is one of the most applied metals in microelectronics as solder and surface finish material due to its good conductivity, corrosion resistance, low cost, and solderability.

The growth of Sn whiskers has become a serious reliability issue in the microelectronic industry since Sn whiskers can grow to the length of a millimeter, causing short circuits, which can be fatal to electronic devices. During the past decades, the growth of Sn whiskers has caused several problems in various applications, as shown in Table 1.

The best mitigation of Sn whiskers was using Sn-Pb alloys. In 1959, Arnold [5] reported that Sn whiskers were mitigated when the Sn plating was alloyed with Pb. In 1990, Cunningham and Donahue [6] of the Raytheon Company compared the Sn whisker growth on reflowed Sn and Sn-Pb layers subjected to mechanical stress at high temperatures. The least whiskers were detected on Sn60Pb40 surfaces. The application of Pb against Sn whiskers was standardized in some companies. Diehl [7] from Bundy Connector Corporation reported that Pb should be added to prevent whisker formation in Sn electroplating, and his guidelines have been used for all Sn-plated connector products from Bundy Corporation till 2006. The alloying of Pb with Sn entirely solved the Sn whisker problem till the lead-free change.

The European Union’s Restriction of the Use of Certain Hazardous Substances in Electrical and Electronic Equipment (RoHS) directives in 2006 required the elimination of lead (less than 0.1 wt% is allowed) from electronic devices by July 2006 [8]. Banning Pb in the solder alloys increased Sn content in the solder alloys by over 95 wt% [9,10], which raised the possibility of Sn whisker growth significantly, requiring new prevention/mitigation methods.

### 1.2. Lead-Free Soldering

Lead-free soldering was introduced due to Pb’s hazardous property. Lead can oxidize to PbO in the atmosphere:

Pb + 1/2 O_2_ → PbO (1)

And, then, when the electronic waste leaches into the soil, it can be dissolved from the solder joints into PbSO_4_ and Pb(NO_3_)_2_ by sulfuric acid (H_2_SO_4_) or nitric acid (HNO_3_) contained in acid rain:

Pb + H_2_SO_4_ → PbSO_4_ + H_2_O (2)

Pb + 2HNO_3_ → Pb(NO_3_)_2_ + H_2_O (3)

The human body can absorb lead and cause adverse health effects, including lead poisoning, decreased intelligence, and reproductive and nervous system damage.

Therefore, additional regulations were introduced worldwide, like Waste from Electrical and Electronic Equipment (WEEE) directives, which stipulate reuse and recycling, since August 2005, or the Home Appliance Recycling Law in Japan since 2001, and the PC Recycling Law and the New Energy Development Organization (NEDO) to further accelerate environmental conservation efforts [11]. These restrictions have also impacted previously commercialized Sn-Pb solder materials. It has to be noted that the RoHS directives regulate not only the use of Sn but also five other hazardous substances (Hg, Cd, Cr^6+^, PBB, and PBDE) [12].

At the beginning of the lead-free change, the first challenge was finding supplementing lead-free alloys with mechanical, electrical, thermal, and structural properties that are at least equal to or better than the Sn-Pb solder. Rather than searching for new base metals, the development of solder alloys based on Sn was started since Sn has good reaction ability with many other metals. It can form intermetallic compounds, having a relatively low melting temperature after alloying [13,14,15,16,17]. Several alloy compositions have been tested, including Sn-Ag-Cu, Sn-Ag-Zn, Sn-Cu, Sn-Ag, Sn-Zn [18], Ag-Bi and Sn-Bi [19], Sn-Ag, Sn-Cu, Sn-Bi, Sn-In, Sn-Zn, Sn-Sb, and Sn-Ge [20].

Of these alloys, Bi-, Ge- and In-containing alloys are too expensive. Zn-containing alloys have poor wettability because Zn is an active element that is prone to oxidization [21]. Sn-Sb alloys have proven to be a better superconductor material than solders [22,23]. Sn-Cu alloys are cheap and have good wettability, but they have high melting points, and, when applied to the wave soldering of double-sided boards, it is difficult for the molten solder to fill the through holes sufficiently [24]. So, finally, the Sn-Ag-Cu alloy (SAC) spread in the industry. A challenging aspect of applying SAC solder alloys was the increased formation of silver and copper intermetallic compounds (IMCs). The IMC layer formation at the interface of the solder and the contact pad is an essential soldering condition for achieving electrical and mechanical connections. However, the Ag and Cu content of the solder alloys also causes the IMC formation inside the solder bulk.

The thermodynamically reactive Ag and Cu react with Sn to form IMCs of Ag_3_Sn and Cu_6_Sn_5_ in the solder bulk. Cu_6_Sn_5_ has a rod-like structure, and Ag_3_Sn has a plate-like structure. Generally, the formation of dispersed Ag_3_Sn IMCs in the solder bulk positively affects the mechanical parameters of the solder joints, so increasing the weight fraction of Ag in the SAC alloys can be beneficial, but not unlimitedly. Over 3.2 wt% of Ag, large Ag_3_Sn platelets could form, which can decrease the thermo-mechanical properties of the solder joints [25]. At the interface of SAC solders and Cu contact surfaces, the increased IMC layer growth is also observed, containing Cu_6_Sn_5_ to the solder balk below Cu3Sn on the Cu layer, according to the following [26,27]:

6Cu + 5Sn → Cu_6_Sn_5_ (4)

3Cu + Sn → Cu_3_Sn (5)

Cu_3_Sn can form only above 60 °C; furthermore, it is thermodynamically unstable at the Cu/Sn interface and dissociates into Cu_6_Sn_5_:

2Cu_3_Sn + 3Sn → Cu_6_Sn_5_ (6)

This results in Cu_3_Sn being relatively thin compared to Cu_6_Sn_5_, with a thickness ratio of usually 1:10–20.

In the case of Ni-plated contact surfaces, Sn can form IMCs with Ni as well. From the solder bulk, a layer of thicker Ni_3_Sn_4_, thinner Ni_3_Sn_2_, and a very fine Ni_3_Sn is formed [28]. The thickness of the whole IMC layer is usually between 1 and 2 µm in an SAC solder joint. Although the IMC layer is an essential condition of the solder joints, it has worse mechanical and electrical parameters than the solder bulk; many quality and reliability problems originate from them, so their increased growth should be avoided. Furthermore, IMC layer growth could be a direct reason for Sn whisker growth.

## 2. Sn Whisker Growth

**Figure 2:**
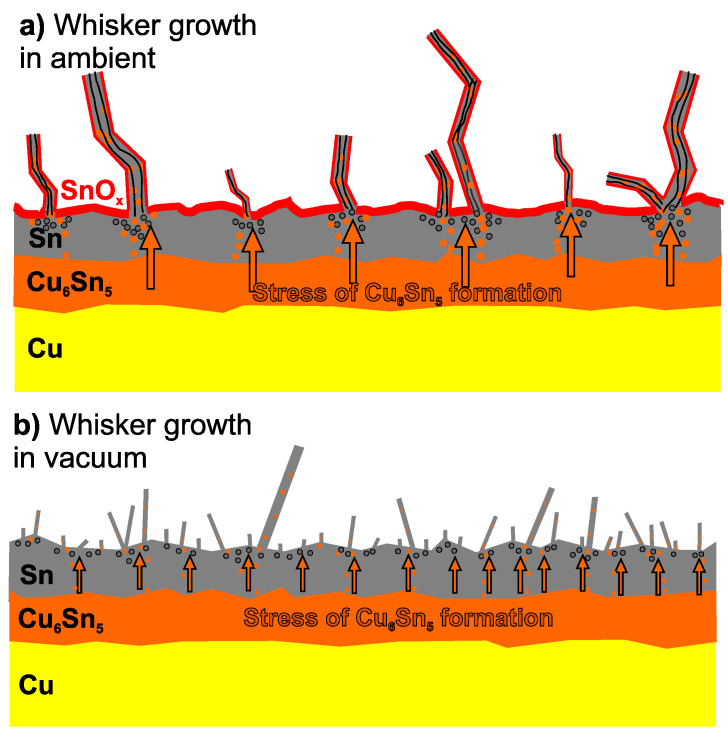
Whisker growth induced by the Cu_6_Sn_5_ IMC layer formation: (**a**) in ambient conditions; and (**b**) in vacuum conditions [35].

Sn whisker growth can occur in lead-free solder alloys [29,30,31,32]. The Sn whisker growth is caused by the development of compressive mechanical stresses within the Sn layer, which can originate from several sources: direct mechanical load, the residual stress of layer deposition, temperature change-induced stress, and volumetric change-induced stress. As shown in Figure 2, the whisker extruded as a stress release mechanism in the case of compressive stress inside the solder layer [33,34,35]. Among the above-listed stress sources, volumetric change-induced stress is the most complex and least avoidable.

The Cu_6_Sn_5_ IMC layer grows into the solder bulk and has a higher density (8270 kg/m^3^) than Sn (7265 kg/m^3^), resulting in volume shrinkage at the Cu-Sn interface. This volumetric change can generate a large mechanical stress on the Sn [28], according to the following [36]:

(7) $$\sigma =(\frac{E_{Cu_{6}Sn_{5}}}{1-\upsilon })\cdot \frac{\Delta V}{V}$$

where *E_Cu_*_6*Sn*5_ is Young’s modulus [Pa], *υ* is the Poisson coefficient, and *V* is the volume [m^3^] because the Young’s modulus of Cu_6_Sn_5_ is large, between 50 and 150 GPa [37,38].

Pei et al. [39] executed in situ measurements with synchrotron micro-diffraction and complementary fluorescence to determine the surface and interface morphology, local microstructure, and deviatoric strain in Sn-Cu systems. Their most interesting finding was that there was no excessive IMC accumulation in the area where the monitored hillock grew. A comparison of the IMC and stress maps showed that the biaxial stress was distributed uniformly across the surface, although IMC grew mostly along the Sn grain boundaries. It means that a locally high compressive stress or high-strain energy density does not necessarily result in whisker or hillock growth. The route of the monitored hillock had a lower biaxial stress and strain energy density than the surrounding areas, which could provide the driving force for the growth.

In contrast, later, Pei and Chason [40] found a direct correlation between stress relaxation and whisker growth. They applied a MOSS system to measure the stress change in the samples during whisker growth in thermal cycling conditions. In all the cycles, the thermal stress built up with the temperature increase before the whiskers started to grow, and the stress began to relax soon after whiskering was observed.

**Figure 3:**
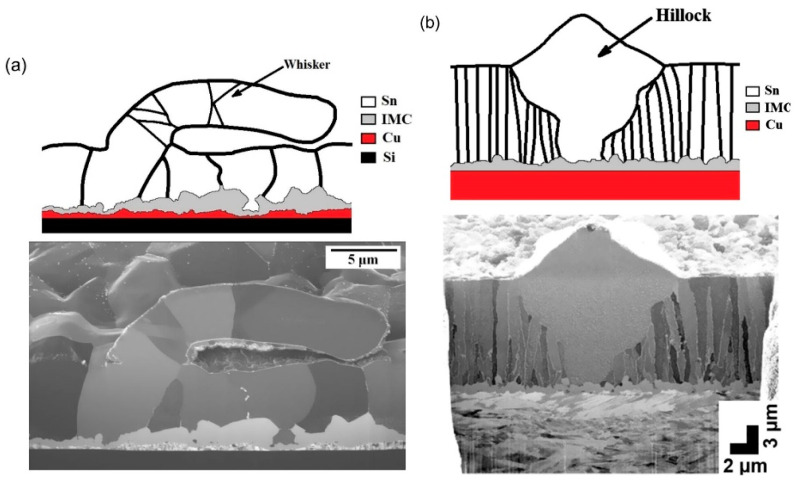
FIB cross-sections of Sn whiskers: (**a**) a nodule whisker grew from a mat Sn layer; and (**b**) a hillock grew from a bright Sn layer [3].

Figure 3 presents a cross-sectional FIB image of the Sn layer, in which the Cu_6_Sn_5_ IMC layer formation at the interface of the electrodeposited Sn and the Cu substrate resulted in Sn whisker growth. The micrographs were created using secondary electrons generated by a focused ion beam, so there is more excellent contrast between different particle orientations than in a typical SEM image. The schematic line drawings above the micrographs are shown to highlight the primary features in the images as discussed in the text [3]. Figure 3a shows the cross-section of a nodule that a whisker grew from the mat Sn layer electrodeposited on a Cu substrate. The mat Sn layer had large Sn grains with a columnar-grain structure. The whisker bent so that its growth direction was almost parallel to the Sn layer. Figure 3b shows the cross-section of a hillock that grew from a bright Sn layer electrodeposited on a Cu substrate. The bright Sn layer had a very fine grain structure.

Volumetric change-induced stress can also be caused by corrosion of the Sn. There are two types of tin oxides: stannic oxide (SnO_2_) and stannous oxide (SnO). The presence of these two oxides reflects the double valence of Sn (oxidation states of 2^+^ and 4^+^, respectively). SnO is not as well characterized as SnO_2_ [41]. They have the following crystal structures: SnO has a tetragonal unit cell with a litharge structure like PbO [42]. SnO_2_ has a tetragonal unit cell [43] and has the same rutile structure as many other metal oxides (e.g., TiO_2_). Osenbach et al. [44] investigated Sn-plated samples tested under corrosive conditions. They found that, in those areas of the Sn layer where moisture condensed, the entire Sn layer corroded fast, and the corrosion spots contained mostly crystalline SnO_2_.

The specific density of Sn is 7310 kg/m^3^, while SnO and SnO_2_ have lower densities, 6450 kg/m^3^ and 6950 kg/m^3^, respectively. Hence, the volumetric increase in the solder bulk could initiate considerable compressive stress. The phenomenon of density reduction is manifested in the structural change in single crystals. In the case of conventional β-Sn, four Sn atoms are contained in one single crystal, but, in the case of SnO_2_ single crystals, two Sn atoms and four O atoms are included, resulting in a decrease in the number of Sn atoms per single crystal. In other words, one Sn single crystal is oxidized to two SnO_2_ single crystals, resulting in a 32.72% increase in volume. Although corrosion may appear only on the top surface of the solder joints, it can spread deep into the solder joint, causing mechanical stress even in the solder bulk. The combination of condensed moisture and high temperature has been shown to produce localized whisker clusters and accelerate the growth of individual whiskers [45]. Excessive Sn atoms due to oxidation are confined within the original volume of the Sn layer, generating local stress and excessive strain energy [41].

Schroeder et al. [46] also showed that whisker growth can be triggered by high temperature and humidity conditions by corrosion. Su et al. [47] conducted a statistical study on the whisker population and growth. They found that higher humidity increased the number and length of whiskers, which means that whisker growth is promoted in environments where corrosion is more likely to occur. The corrosion resistance of the solder alloy, the expansion rate of corrosion products in the solder bulk, and the recrystallization rate of the solder alloy can considerably vary the corrosion-induced whisker growth susceptibility from the different solder alloys [48].

Beyond the inducing effect of the SnO_x_ formation on whisker growth, it has a further important aspect, namely, without a surface oxide (SnO_x_) layer, the internal compressive stress in the Sn is relaxed uniformly across the Sn surface, inhibiting or at least suppressing whisker growth. In the presence of the SnO_x_ layer, the internal compressive stresses in the Sn layer can be relaxed only at specific weak points of the SnO_x_ layer where the Sn whiskers can start to grow [49].

## 3. Suppressing Whisker Growth in Lead-Free Alloys

### 3.1. Alloying

**Figure 4:**
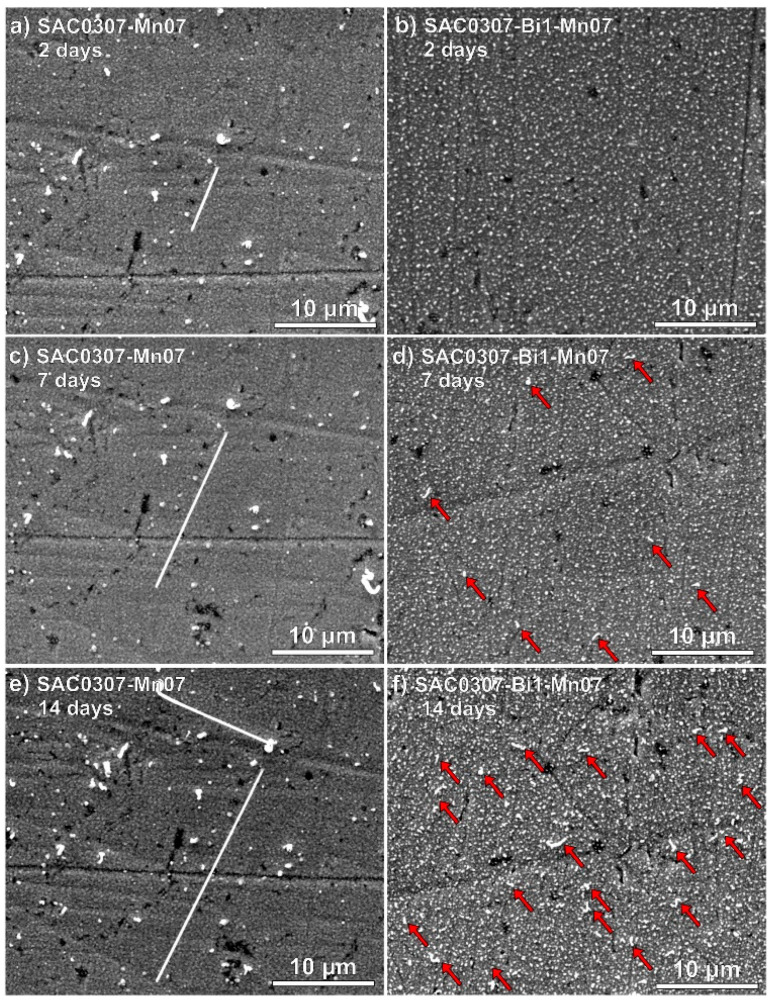
Sn whiskers on the different samples after some days of the layer deposition: (**a**) SAC0307-Mn07, 2 days; (**b**) SAC0307-Bi1-Mn0.7, 2 days; (**c**) SAC0307-Mn0.7, 7 days; (**d**) SAC0307-Bi1-Mn0.7, 7 days; (**e**) SAC0307-Mn0.7, 14 days; and (**f**) SAC0307-Bi1-Mn0.7, 14 days [50]. (Red arrows indicate smaller whiskers on the surface).

Based on the suppressing effect of Pb on Sn whisker growth, even from a relatively low amount (>3% Pb) [30], it seemed a straightforward solution to improve the lead-free alloys’ whisker susceptibility by alloying further metals into the solders. Bismuth (Bi) has similar physical properties as Pb, and it was found that Bi can also be effective in suppressing whickering, even in a low amount of 2–4 wt.% [9]. Illés et al. [50] investigated the Sn whisker growth from SAC0307-Mn0.7 and SAC0307-Bi1-Mn0.7 film layers made by PVD on copper substrate. They observed that, at room conditions, the SAC0307-Mn0.7 developed whiskers right after the sample preparation, while the SAC0307-Bi1-Mn0.7 performed whiskers only three days later. Furthermore, Bi content almost suppressed the growth of long filament whiskers (Figure 4).

They found that the Bi formed separated gains in the layer, which decreased the creep resistance of the SAC-Mn alloy. The higher creep resistance of the SAC-Mn alloy resulted in an easy transfer of mechanical stress from the bulk layer to the surface defects, which formed the roots of the whiskers. The more creep-prone SAC-Mn-Bi alloy could accommodate the stress within the more ductile layer. The evenly distributed, softer Bi grains in the Sn lattice also could aid in relieving the mechanical stress in the layer locally by short Bi whisker formation (Figure 4) [50].

The effect of Bi on the mechanical properties of SAC solder alloys and, via this, their Sn whisker susceptibility was the focus of many other research studies. El-Daly et al. [51] tested a high Sn content Sn1.5Ag0.7Cu-1Bi SAC alloy. The Bi content modified the original microstructure of SAC157 solder alloy: β-Sn grains were surrounded with a large number of uniformly distributed small needle-like Ag_3_Sn and Cu_6_Sn_5_ grains. The microstructural refinement blocked the dislocation movements and increased the creep resistance of Bi-containing solder joints. Ali et al. [52] studied the microstructure and tensile properties of SAC105 solder alloys containing 1–2 wt% Bi. They found that Bi can improve the shear strength of solder joints. Mahdavifard et al. [53] found that Bi content reduces the activity of Sn, which is involved in chemical reactions of Ag–Sn and Cu–S, and causes almost constant tensile strength and yield strength over time. The above-discussed effects of Bi on the SAC solder joints might be the reason for whisker growth suppression, as was observed by Illés et al. [50].

**Figure 5:**
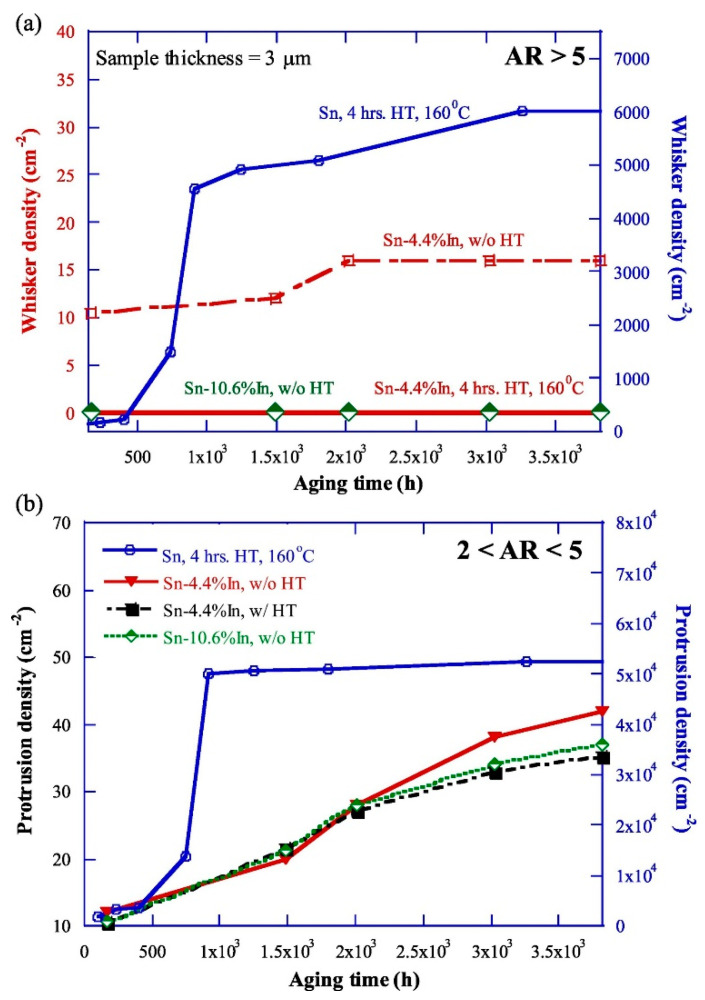
Effect of In doping on the whisker growth from electroplated: (**a**) Sn whisker density on the Sn-In samples; and (**b**) protrusion density on the Sn-In samples [55].

**Figure 6:**
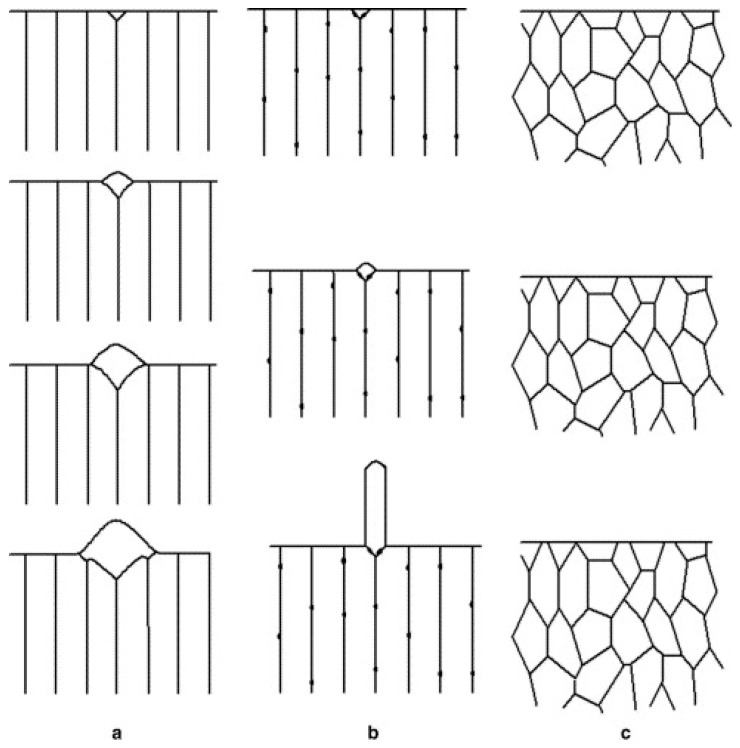
Grain structure of SnCu and SnPb layers: (**a**) hillock growth from a columnar grain structure of SnCu; (**b**) whisker growth from a columnar grain structure of SnCu; and (**c**) the equiaxed structure of SnPb where uniform creep is possible [36].

Other metals like In, Ni, Zr, Cr, or Fe proved positive effects on the microstructural, mechanical, or chemical properties of Sn-based solder joints and layers, which could suppress the Sn whisker growth. Mahapatra et al. [54,55] have found that electroplated Sn doped with some In might effectively inhibit whisker growth during long-term high-temperature (160 °C) storage. Figure 5 shows that 4.4 wt% of In in the Sn layer almost eliminated the Sn whiskers, and, over 10 wt% of In in the Sn layer, Sn whiskers did not appear at all. OES and XPS measurements proved that In was concentrated on the surface of the Sn layer, and In could suppress the Sn layer’s oxidation by including In in the surface Sn oxides (Figure 6b). The separation of In atoms at the grain boundary reduced the stiffness of the oxide film and changed the stress gradient that suppressed the growth of whiskers by reducing the grain boundary self-diffusivity of Sn [54,55].

Yao et al. [56] investigated the effect of Ni alloying on the Sn3.8Ag0.7Cu solder and found that the Ni particles react with Sn in the solder and form Ni-Cu-Sn IMCs. When Ni exceeded 1 wt%, only boomerang-shaped (NiCu)_3_Sn_4_ was found at the solder joints’ interface. In addition, the Cu content of the IMC decreased as the Ni content increased, and the shear strength of the solder joints increased continuously. Lu T et al. [57] studied mixing Zr into SAC305 at weight fractions between 0, 0.05, 0.2, and 0.5 wt%. Adding Zr resulted in a 59.6% reduction in the size of β-Sn grains. The morphology of Ag_3_Sn IMCs changed from strip to dot, and ZrSn_2_ IMCs were also found in the solder bulk. The elongation and shear strength improved up to 0.2 wt% of Zr, but both properties decreased over it.

Similar effects to Ni were observed when Fe particles were added to the Sn3.0Ag0.5Cu solder alloy; Fe core particles with an outer FeSn_2_ reaction layer were observed [58]. Although Co can also form CoSn_2_ IMCs with Sn, which dispersed along the interdimeric region, no positive effect was observed in the case of Co alloying into SAC solders [59]. Adding Cr into SAC solder joints transformed the long needle-like Ag_3_Sn into small and refined Ag_3_Sn particles, resulting in mechanical improvements of the alloy [44].

Unfortunately, some metals can have a negative effect on whisker susceptibility. Wu et al. [60] added rare earth Pr (~0.06 wt%) to the SAC0307 solder to enhance its wettability, shear force, and ductility. However, the addition of Pr led to the formation of the PrSn_3_ phase, and they became the roots of the Sn whiskers since their development caused compressive stress. Later, they [61] compared the Sn whisker growth from the SAC307-1Pr solder under different environments to study the effects of temperature, oxygen level, and NaCl on whisker growth. They proved that temperature and oxygen level are the most important factors that affect Sn whisker growth since these factors determine the oxidation rate of the PrSn_3_ phase as the main contributing factor for whisker growth. Zhang et al. [62] doped the Sn3.8Ag0.7Cu solder with Ce between 0 and 1.8 wt% to improve its wettability, tensile strength, and thermal fatigue behavior. They observed that the doping of Ce over 0.1 wt% considerably increased the whisker susceptibility of the solder alloy, even in room conditions. It was supposed that the higher chemical potential of Ce for oxidation enhanced the whisker growth. Chuang [63] observed rapid whisker growth on the surface of SAC305-1.0Ce solder joints caused by the formation of CeSn_3_ phases.

SnCu alloys are more prone to whisker growth than pure Sn [64]. As it was discussed above, in the SnCu alloys, the Cu forms Cu_6_Sn_5_ IMCs in the solder bulk. Williams et al. [65] showed that adding Cu to a plating electrolyte can form Cu_6_Sn_5_ IMCs along the Sn grain boundaries and cause residual stress in the layer. Boettinger et al. [66] found that, in a SnCu layer on a Cu substrate, a columnar grain structure is forming (Figure 6a,b), which cannot relax the compressive stress generated by Cu_6_Sn_5_ IMC layer formation (Figure 6a,b). In the SnCu layer, the in-plane compressive stress is relieved by hillock or whisker growth, depending on whether the grain boundaries are mobile (Figure 6a) or pinned (Figure 6b) due to the lack of transverse grain boundaries in the columnar grain structures. Figure 6c represents the grain structure in a SnPb layer, which had an equiaxed structure without columnar grains, where the internal grain boundary is effective in stress relaxation. So, the inclusion of Pb changes the crystal structure of Sn, which has an inhibiting effect on whisker growth [36].

### 3.2. Composite Solder Alloys

The latest development direction of soldering is the composite solder alloys [66,67], which means the addition of carbon-based ceramics [68,69,70] or metal oxide nanoparticles [71] into the solder alloys. The usual weight friction of the nanoparticles is between 0.1 and 1 wt%. The nanoparticles can be added in three different ways to the solders: simply mixing the nanoparticles into the solder paste (mostly applied), adding them during the alloying process, or mixing them with the solder balls before solder paste preparation (powder metallurgy) [72]. Most popular ceramics are as follows: TiO_2_, ZnO, SiC, Al_2_O_3_, ZrO_2_, Si_3_Ni_4_, ZrO_2_, Fe_2_O_3_, etc. [67,73,74,75]. The nanoparticles usually have advantageous effects on the mechanical properties of the composite solder joints [76,77], with some change in melting temperature (±1–2 K) [78].

The nanoparticles are non-soluble in tin, which results in their scattering in the Sn-matrix, and they are incorporated at the grain boundaries of Sn and IMC grains. Furthermore, the nanoparticles enhance the heterogeneous nucleation of the grain growth during the solidification of the solder alloy, which refines the Sn and IMC grains and decreases the dislocation motion [79,80,81]. The microstructural changes are responsible for the mechanical improvements in the composite solder joints, which could positively affect the whisker susceptibility of the solder alloys, as the above discussed other alloying elements.

Ma et al. [82] studied the inhibition of whisker growth using POSS-silanol. Previous studies [83] and [84] reported that adding 3 wt% POSS-silanol to SAC305 solder improved the shear strength and microhardness of SAC305 solder by 42.86% and 11.18%, respectively. Furthermore, POSS-silanol inhibited the growth of interfacial IMC. Therefore, the same amount of 3 wt% Poss-silanol was mixed with SAC305 and subjected to thermal cycling at −45 to 85 °C for reliability [82]. The results showed that the added POSS-silanol made the composite solder matrix harder and significantly inhibited the formation of whiskers and interfacial deformation under thermal cycling conditions.

**Figure 7:**
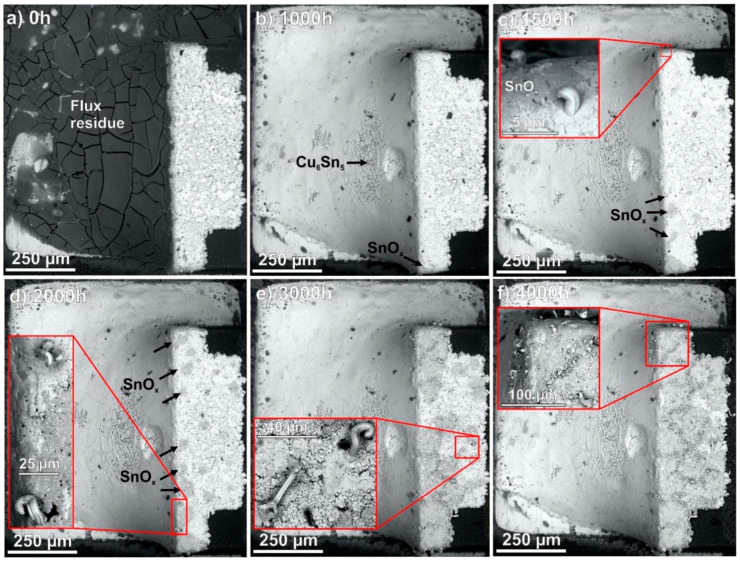
SEM micrograph of a reference SAC0307 solder joint during the TH test: (**a**) at 0 h; (**b**) at 1000 h; (**c**) at 1500 h; (**d**) at 2000 h; (**e**) at 3000 h; and (**f**) at 4000 h [77].

**Figure 8:**
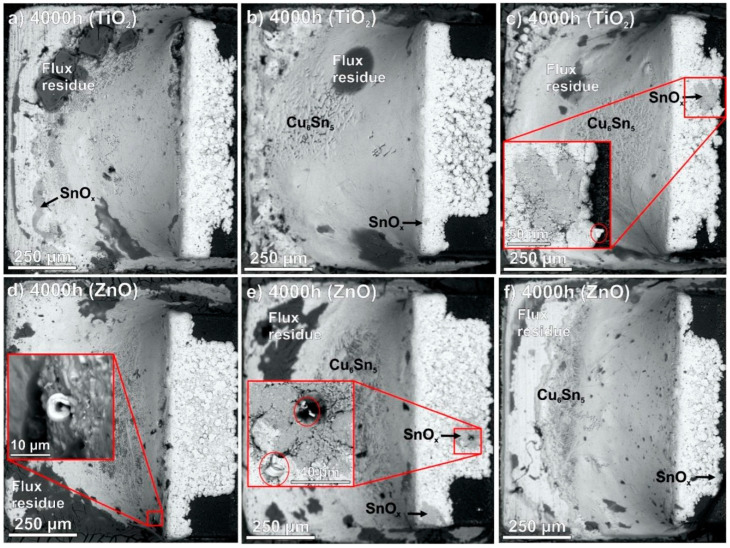
SEM micrographs of SAC0307-TiO_2_ (0.25 wt%) and SAC0307-ZnO (0.25 wt%) composite solder joints after a 4000 h 85 °C/85RH% test: (**a**) TiO_2_ (1); (**b**) TiO_2_ (2); (**c**) TiO_2_ (3); (**d**) ZnO (1); (**e**) ZnO (2); and (**f**) ZnO (3) [85].

Illés et al. [85] studied the growth of Sn whiskers from composite solder joints of SAC0307 (Sn0.3Ag0.7Cu) with TiO_2_ and ZnO nanoparticles. They conducted a thermal-humidity test for 4000 h at 85 °C/85 RH%. As shown in Figure 7, the growth of Sn whiskers was observed in SAC0307 after 1500 h. At the end of the thermal-humidity test, the solder joints were covered with numerous whiskers. However, in the composite solder joint with a 0.25 wt% of TiO_2_ and ZnO nanoparticles, only slight corrosion traces were found after 4000 h of the thermal-humidity test, as shown in Figure 8. A few short whiskers grew around the corrosion spots, but they appeared only at 3500 h. It was found that TiO_2_ and ZnO nanoparticles are incorporated between the Sn grains, and they can bond to the Sn atoms with high binding energy and form a protective oxide layer, which could block the spread of corrosion and ultimately prevent the growth of Sn whiskers [85].

**Figure 9:**
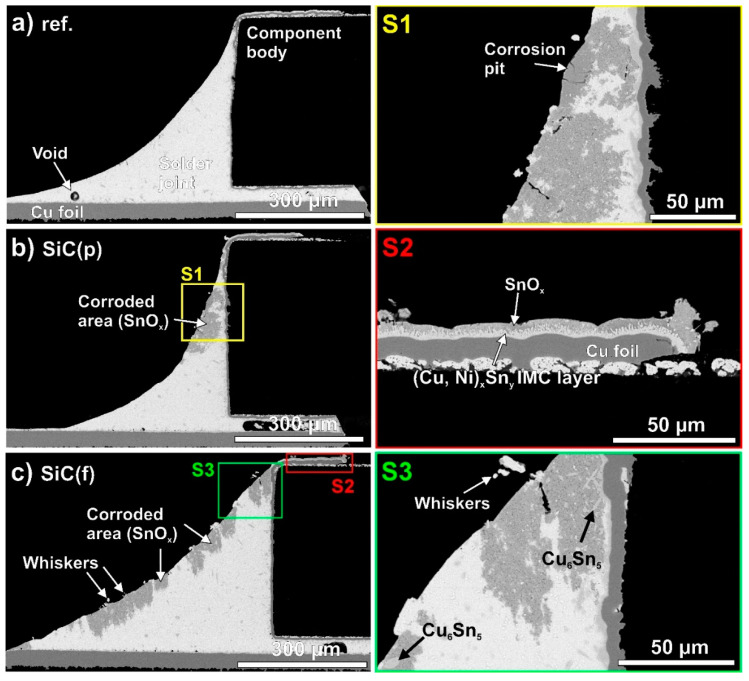
SEM micrographs of cross-sectioned solder joints: (**a**) ref. SAC0307; (**b**) SAC0307-SiC(powder); and (**c**) SAC0307-SiC(fiber) [86].

Unfortunately, some other ceramic nanoparticles have the opposite effect on Sn whisker growth. Choi et al. [86] investigated the corrosion reliability of SAC0307-0.5SiC composite joints, and they found that the SiC nanoparticles considerably decreased the corrosion resistance and caused the growth of the double amount of Sn whiskers on the composite joints than on the reference SAC0307. Figure 9 presents cross-sectioned solder joints after 4000 h of an 85 °C/85 RH% corrosive test. Corrosion spots with a large extent and deeply penetrated into the solder bulk were found at the composite joints (Figure 9b,c). It was found that the SiC can bond to the Sn atoms, like TiO_2_, but SiC is prone to corrosion and acts like corrosion–incubation points at the Sn grain boundaries [86].

**Figure 10:**
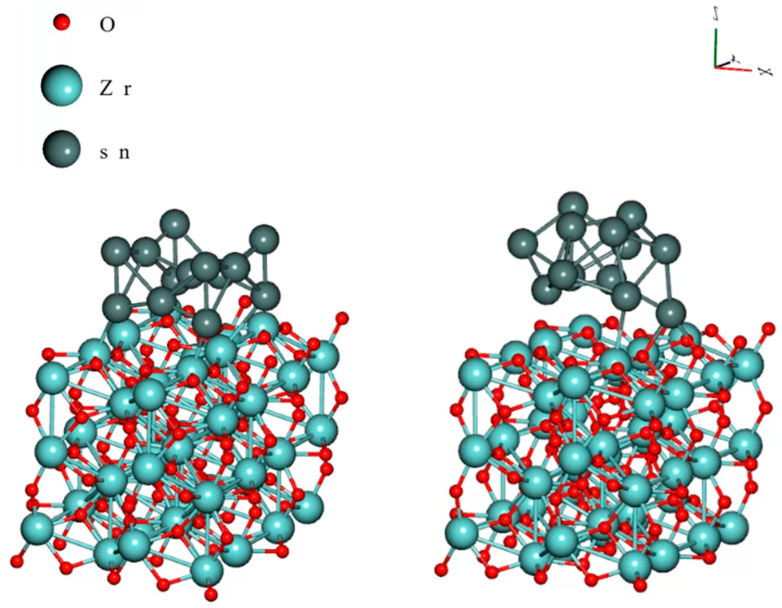
DFT simulation of the Sn cluster on the ZrO_2_ crystal, the initial position is **left**, and the optimized position is **right** [88].

Similar results were observed by Illés et al. [87] during the reliability testing of SAC0307-0.5CuO solder joints. CuO nanoparticles enhanced the corrosion and the whisker growth from the composite solder joints. They found that CuO can react with dispersed Cu_6_Sn_5_ IMCs in the solder bulk, and the reaction results in the formation of even SnO_2_. Skwarek et al. [88] investigated the effect of ZrO_2_ nanoparticles in SAC0307 solder and also observed enhanced corrosion, but the correlation between the extent of the corroded area and the intensity of Sn whisker growth was weaker than in the previous cases. DFT simulations (Figure 10) proved that ZrO_2_ can also bond to Sn, but the binding energy is relatively low, only 1.15 eV, compared to TiO_2_ (2.12 eV) [88].

It was postulated that, generally, a refined grain structure caused by doping the nanoparticles decreases the corrosion resistance since the corrosion typically starts at the grain boundaries, where the surface free energy is higher. The grain refinement causes an extensive grain boundary network in the composite solder joints and increases the grain-boundary free energy of the system. So if the binding between the nanoparticles and the Sn atoms is not strong enough (like in the case of ZrO_2_ and Sn), then the nanoparticles cannot form the corrosion protection layer at the grain boundaries [88] (as the TiO_2_ and ZnO could [85]).

Wu et al. [89] improved the wettability of the composite SAC0307-Al_2_O_3_ solder by applying a precoating surface on the Al_2_O_3_ nanoparticles with rare-earth (RE) Pr. They found the optimum weight fraction of the dopants at 0.06 wt% when the composite SAC0307-0.06Pr-0.06Al_2_O_3_ performed the best wettability, a well-controlled growth of interfacial IMC layer, the highest shear force (∼61 N) and superior creep resistance. However, the observed appearance of the Sn whisker was near the SAC0307-0.06Pr-0.5Al_2_O_3_ solder/Cu interface.

Generally, the most important and sensitive parameter of composite soldering is the amount of nanoparticles in the solder joints. Usually, there is an optimal amount where the nanoparticles can provide their best positive effects on the solder joints; below the optimal wt%, effects are negligible; over the optimal wt%, the agglomeration of the nanoparticles can even cause the degradation of the joints. Illés et al. [90] found that, over 0.5 wt% of TiO_2_ nanoparticles in SAC0307, TiO_2_ agglomerated and could not inhibit the growth of Sn whiskers, as it was observed at 0.25 wt%. Nai et al. [91,92] used multi-walled carbon nanotubes (MWCNTs) by powder metallurgy in the SAC357 solder. They found that composite solder joints had better mechanical properties at 0.01 wt%, but adding more MWCNTs caused numerous microporosities in the solder joints, which reduced their mechanical properties [92]. Mao et al. [93] doped the Sn0.7Cu solder with Ni-coated CNTs in 0, 0.025, 0.05, 0.075, 0.1 wt%. They observed mechanical improvements only still 0.05 wt% over the degradation detected. Rajendran et al. [94] added ZnO nanoparticles into the SAC305 solder alloy between 0 and 0.7 wt% and found that, over 0.2 wt%, the ZnO considerably decreased the wetting ability of the composite solder; however, it did not decrease the mechanical parameters of the solder joints.

Table 2 summarizes the effects of the different alloying elements and nanoparticles on the Sn-based solders.

## 4. Conclusions

After the restriction of Pb in solder alloys due to environmental issues, the reliability issues of Sn whisker growth are re-emerging. Several mitigation methods, including alloying and reinforcement of the solders, were studied to prevent or at least suppress the Sn whisker growth. Generally, modifying the solder composition is a promising method to decrease the reliability risk caused by Sn whisker growth in microelectronic devices. Most of the additional alloying metals suppress/refine the IMC formation in the solder bulk and at the interface between the substrate and the solder joint and/or modify the Sn grain structure, the effects of which can decrease the whisker susceptibility. The best results were achieved with alloying Bi and In.

The results of the reinforced composite solders are controversial. POSS-silanol can be effective against IMC-induced whisker growth, such as alloying. Adding TiO_2_ and ZnO nanoparticles could considerably increase the corrosion resistance of the composite solder joints and, via this effect, suppress the corrosion-induced whisker growth. However, SiC, CuO, and ZrO_2_ nano-particles affected oppositely the whisker susceptibility of the composite solder joints. The amount of the reinforcement is also a key factor since, if it exceeds a certain level, it may not inhibit the growth of Sn whiskers.

We can state that the mechanism of Sn whisker growth is fully understood, but the elimination has not been fully solved yet; the continuation of extensive research is still necessary. Promising future research directions could be the following: (i) search further alloying elements that can positively affect the mechanical parameters of the solder alloys; (ii) investigate the whisker susceptibility of the low-temperature BiSn and BiSnAg solder alloys, which have become more and more popular in the past years; and (iii) continue to study the whisker suppression effects of TiO_2_ and ZnO nanoparticles.

## Figures and Tables

**Table 1:** Famous Sn whisker-caused reliability issues, 1986–2003 [4].

Year	Application Title 2	Sector Title 3	Whisker Place
1986	Heart Pacemakers	Medical	Crystal Can
1986	MIL Aircraft Radar	Military	Hybrid Package Lid
1987	MI:/Aerospace PWB	MIL/Aerospace	PCB Traces
1988	Missile Program “B”	Military	Electronic Enclosure
1993	Govt. Electronics	Govt. Systems	Transistor
1998	Commercial Satellite	Space	Relays
2000	Missile Program “D”	Military	Terminals
2000	Power Mgmt. Modules	Industrial	Connectors
2001	Hi-Rel	Hi-Rel	Ceramic Chip Cap
2002	Electric Power Plant	Power	Microcircuit Leads
2002	GPS Receiver	Aeronautical	RF Enclosure
2003	Telecom Equipment	Telecom	Ckt Breaker

**Table 2:** Effects of the alloying elements and nanoparticles on the Sn-based solders.

Element	Technology	Effect	Reference
Pb	alloying	Suppressing whisker growth; decreasing the melting temperature	[5,6,7,28,30]
Ag	alloying	Neutral effect on whisker growth; forming Ag_3_Sn IMC, no effect on whiskers	[18,19,20,25]
Cu	alloying	Forming Cu_6_Sn_5_ (one root cause of whisker growth) and Cu_3_Sn IMCs	[18,19,20,26,27,28,36,37,38,39]
Ni	alloying	Neutral effect on whisker growth; forming Ni_3_Sn_4_, Ni_3_Sn_2_, and Ni_3_Sn IMCs; increased shear strength	[56]
Bi	alloying	Suppressing whisker growth; decreasing the melting temperature, and improving mechanical parameters	[50,51,52,53]
Mn	alloying	Neutral effect on whisker growth; increasing the melting temperature	[50]
In	alloying	Suppress whisker growth, decreasing the melting temperature	[54,55]
Zr	alloying	Neutral effect on whisker growth; reducingβ-Sn grains	[57]
Fe	alloying	Neutral effect on whisker growth; forming Fe_2_Sn reaction layer	[58,59]
Co	alloying	Neutral effect on whisker growth; forming CoSn_2_ IMC	[59]
Cr	alloying	Neutral effect on whisker growth; some mechanical improvements	[44]
Pr	alloying	Enhancing wettability, shear force, and ductility; forming PrSn_3_ causes whisker growth	[60,61]
Ce	alloying	Forming CeSn_3_ and oxidation causes whisker growth	[62,63]
POSS-silanol	nanoparticles	Suppressing whisker growth; improving the mechanical parameters	[82,84]
TiO_2_	nanoparticles	Suppressing whisker growth; improving the mechanical and corrosion parameters	[74,77,80,85,90]
ZnO	nanoparticles	Suppressing whisker growth; improving the mechanical and corrosion parameters	[76,85,94]
SiC	nanoparticles	Enhancing whisker growth; improving the mechanical but decreasing the corrosion parameters	[86]
CuO	nanoparticles	Enhancing whisker growth; improving the mechanical but decreasing the corrosion parameters	[87]
ZrO_2_	nanoparticles	Enhancing whisker growth; improving the mechanical but decreasing the corrosion parameters	[67,69,71,88]
Al_2_O_3_	nanoparticles	Enhancing whisker growth; improving the mechanical and wetting parameters	[27,89]
MWCNT	nanoparticles	Neutral effect on whisker growth; improving the mechanical parameters	[91,92]
CNT	nanoparticles	Neutral effect on whisker growth; improving the mechanical parameters	[93]

## Data Availability

Not applicable, no data were used during this study.
